# Clinical and Epidemiological Study of Poisoning Cases Presenting to the Emergency Department of a Tertiary Care Center in Central India

**DOI:** 10.7759/cureus.52368

**Published:** 2024-01-16

**Authors:** Sanjay Samaria, Vinay Pandit, Swapnil Akhade, Subhabrata Biswal, Pankaj K Kannauje

**Affiliations:** 1 General Medicine, All India Institute of Medical Sciences, Raipur, Raipur, IND; 2 Forensic Medicine, All India Institute of Medical Sciences, Raipur, Raipur, IND; 3 Internal Medicine, All India Institute of Medical Sciences, Raipur, Raipur, IND

**Keywords:** overdose, pesticide, emergency, toxicology, organophosphorus, epidemiology, poisoning

## Abstract

Objective

To analyze the clinical and epidemiological characteristics of acute cases of poisoning and the pre-hospital measures that the patient receives before seeking care in an emergency department at a tertiary care center in Central India.

Methods

An observational prospective study was carried out over 18 months, and the relevant findings were documented using a predesigned data collection form. All patients who presented to the emergency department and were 18 years of age or older were recruited, and consent was sought. Data analysis was performed using the SPSS software.

Results

A total of 102 patients diagnosed with poisoning were taken for this study, and data were collected and analyzed. The mean age was 32.8 ± 13.75 years. Of the study population, 63 (61.8%) patients were males. In our study, the most common cause of poisoning was impulsive intake of poison (n = 22, 21.5%) and suicidal ingestion in patients with depression (n = 18, 17.6%). In the emergency department, 61 patients (59.8%) received gastric lavage, and 37 patients (36.3%) received an antidote. The most common agent of poisoning was pesticide ingestion, accounting for 45 (44%) of the total cases. Prescribed drugs were the second-largest group (n = 19, 18.6%). Other common poisoning agents were rodenticides (n = 12, 11.7%), corrosives (n = 8, 7.8%), and aluminum phosphide (n = 3, 2.9%). Out of 102 patients, 82 patients survived, 15 patients died, and five patients left against medical advice (LAMA). One patient had residual comorbidity and was discharged with jejunostomy. The maximum mortality (22.5%) was due to organophosphorus compounds.

Conclusions

While accidental encounters are also common, intentional self-harm accounts for the majority of poisonings; homicidal motives are less likely. Pesticides were the most often used poisoning agents, followed by prescribed and over-the-counter drugs, rodenticides, corrosive agents, and aluminum phosphide. Of the poisoned cases, 69.6% had a full recovery, 22.54% of them died, and eight (7.84%) among them left against medical advice (LAMA). Organophosphorus chemicals were the cause of the highest mortality (22.5%).

## Introduction

Poisoning is one of the leading causes of morbidity and mortality globally and in India [1]. Poison is a substance that is capable of causing the illness or death of a living organism when introduced or absorbed [2]. Over the past decade, poisoning has become an increasing cause for concern not only in India but globally [3]. In developed countries, the rate of mortality from poisoning varies only from 1 to 2%, but in developing countries like India, it varies between 15 and 30% and is the fourth most common cause of mortality, especially in rural India [4]. The WHO reports suggest pesticides are now the most common method of suicide worldwide. The suicide mortality rate per 100,000 population in 2016 was 16.5, while the global average was 10.5 per 100,000. The most vulnerable are 15- to 29-year-olds, the elderly, and people with special needs [5,6].

Pesticide poisoning in India is highly prevalent due to the widespread use of pesticides for agricultural and household activities. In India, pesticide (mainly organophosphate) poisoning is the leading method of suicide in both men and women aged 15 years or older, corresponding to about 92,000 deaths annually [1]. WHO classifies pesticides based on the toxicity of the technical compound and its formulations. The classification is based primarily on the acute oral and dermal toxicity of the rat since these determinations are standard procedures in toxicology [7].

Other poisoning agents include household agents, envenomation, and drugs. As per a meta-analysis of the prevalence of poisoning from 2010-2020, pesticides were the main cause of poisoning in adults, with an incidence of 63% [8]. The second most common cause of poisoning was miscellaneous agents, followed by prescribed and over-the-counter drugs. The estimated prevalence of pesticide poisoning in Central, East, North East, North, South, and West India was 59.2%, 38.5%, 46.9%, 79.1%, 65.9%, and 53.1% [8]. It is observed that agricultural or household pesticides and drugs are taken intentionally, whereas intake of corrosives, kerosene, and animal bites happens accidentally [9,10].

The history, physical examination, and routine and toxicologic laboratory evaluations are used to establish and confirm the diagnosis of poisoning. The history, although intuitively the source of the most helpful information for identifying the etiology of poisoning, is often unreliable when provided by a patient following intentional ingestion [11,12]. The patient's ability to provide a reliable history is often impaired by direct drug effects or psychiatric illness [13].

There is a need for more data regarding the epidemiological characteristics of poisoning cases in Central India owing to the heavy socioeconomic burden that poisoning imposes. Through our study, we primarily aim to study the clinical presentations and epidemiology profile of the cases of poisoning. Additionally, the study will also analyze the pre-hospital measures that were administered to the patients and the outcome. Through this study, we aim to provide insight into the current burden of the problem that will aid decision-making regarding the necessary corrective measures that are urgently needed in the community.

## Materials and methods

Study characteristics

This was a prospective observational study conducted in the emergency department setting of All India Institute of Medical Science (AIIMS), Raipur in Central India. The inclusion criteria for the study population were all patients who presented to the emergency department with a history and clinical features suggestive of poisoning who were 18 years of age or older. All patients from May 2020 to November 2021 (18 months) were included by consecutive sampling and only excluded if they did not provide consent.

Data collection

Data were collected only after consent, and all necessary resuscitative measures were administered. Medico-legal formalities were carried out as per the institute’s protocol. Data collection was aided by a data collection form, and the details were collected either from the patient directly or from relatives. This included demographic parameters including age, gender, occupation, income, geographical region, marital status, details regarding the poisoning such as type of poison, quantity consumed, place of consumption, the motive behind consumption, details of pre-hospital measures given such as first aid, consulting a local doctor and taking treatment, mode of arrival to the hospital, and time delay to arrive at our center. For all the admitted cases, the length of the hospital stay, any complications during hospital stays, and the condition of the patient at the time of discharge were recorded.

The study protocol conformed to the standard ethical guidelines. Ethical clearance was obtained (AIIMS/RPR/IEC/2020/531) from the Institute’s Ethics Committee before the initiation of the study.

Data analysis

The distribution of data on categorical variables such as gender, demographic profile, pre-hospital measures, and clinical outcome was expressed as frequency and percentage. The association of categorical variables in relation to clinical and demographic profile and clinical outcome was carried out using the chi-square test or Fisher's exact test.

Continuous variables like age and length of hospital stay were expressed as mean (SD) or median (range). The comparison of continuous data in relation to the clinical and demographic profile and outcome was performed using an independent student’s t-test/Mann-Whitney's U test or one-way analysis of variance/Kruskal-Wallis test (based on relevance and appropriateness).

Independent factors associated with the clinical outcomes were carried out by using binary logistic regression or multi-nominal logistic regression based on the number of outcomes. All statistical analyses were carried out at a 5% level of significance, and a p-value <0.05 was considered significant.

## Results

The data were tabulated in Microsoft Excel (Microsoft Corporation, Redmond, Washington, United States) and analyzed with IBM SPSS Statistics for Windows, Version 24 (released 2016; IBM Corp., Armonk, New York, United States). An independent t-test was used to compare the continuous variables, and a chi-square test was used to compare the categorical variables. The p-value ≤0.05 was considered statistically significant.

As shown in Tables 1 and 2, a total of 102 patients diagnosed with poisoning were taken for this study, and data was collected. The mean age was 32.8 ± 13.75 years. Of the study population, 63 (61.8%) were males and 39 (38.2%) were females, and the difference in the distribution is statistically significant (p<0.017). Most of the individuals were in the young age group of 18 to 27 years (51%). The second most common age group was 28 to 37 years old.

**Table 1:** Basic characteristics of the study population The statistical measure used for age is the mean with standard deviation, and for gender and socioeconomic status, it is the proportion.

S.N	Parameter		n=102	P value
1.	Age (years)	Mean ± SD	32.8 ± 13.75	
		Range (min-max)	18-72	
		Male	34.2±12.2	0.207
		Female	30.5 ±10.5	
2	Gender N (%)	Male	63 (61.8%)	0.017
		Female	39 (38.2%)	
3	Socioeconomic status N (%)	Upper	06 (5.8%)	0.007
		Middle	49 (48.1%)	
		lower	47 (46.1%)	

**Table 2:** Age-wise distribution of the study population

Age group (years)	Male (n=63)	Female (n=39)	Total (n=102)	P value
18-27	31 (30.4%)	21 (20.5%)	52 (51%)	0.621
28-37	9 (8.8%)	9 (8.8%)	18 (17.6%)	
38-47	9 (8.8%)	4 (3.9%)	13 (12.7%)	
48-57	9 (8.8%)	3 (2.9%)	12 (11.8%)	
>58	5 (4.9%)	2 (1.9%)	7 (6.9%)	
Total	63 (61.8%)	39 (38.2%)	102 (100%)	

Among patients with poisoning, the majority belonged to an urban area (n = 67, 65.7%), and the difference in the distribution is statistically significant (p<0.05). Table 3 describes the distribution of the study population according to socioeconomic status using the modified Kuppuswamy criteria. Most of the individuals belonged to the middle class (n = 49, 48%), particularly the lower middle (n = 27, 26.4%). The second-largest group was the lower class (n = 47, 45.9%), with subsets of upper lower class (n = 26, 25.4%), and lower class (n = 21, 20.5%), and the difference in the distribution is statistically significant (p = 0.007).

**Table 3:** Distribution of the study population according to socioeconomic status

Socioeconomic status	No. of patients (n=102)	P value
Upper	06(5.8%)	0.007
Upper middle	22(21.5%)	
Lower middle	27(26.4%)	
Upper lower	26(25.4%)	
Lower	21(20.5%)	

Table 4 shows that 58 (56.8%) of patients were married, 41 (40.2%) were unmarried, two (1.9%) were divorced, and one (0.9%) were widowed, and the difference in the distribution was statistically significant (p<0.001).

**Table 4:** Marital status of patients with poisoning

Marital status	No. of patients	P value
Married	58 (56.8%)	<0.001
Unmarried	41 (40.2%)	
Divorced	2 (1.9%)	
Widower	1 (0.9%)	
Total	102	

As shown in Table 5, the maximum number of patients were students (n = 23, 22.5%) and homemakers (n = 21, 20.5%). Other common occupations were laborers (n = 12, 11.8%) and farmers (n = 9, 8.8%).

**Table 5:** Occupational distribution

Occupation	No. of patients
Student	23
Homemaker	21
Laborer	12
Farmer	9
Unemployed	5
Shopkeeper	5
Computer Operator	3
Salesman	2
Self-employed	2
Technician	2
Auto driver	2
Accountant	2
Nurse	2
Clerk	1
Driver	1
Tailor	1
Painter	1
Service Manager	1
Pharmacist	1
Business	1
Police	1
Cook	1
Electrician	1
Carpenter	1
Factory Supervisor	1
Total	102

In our study shown in Table 6, 31 (30.4%) patients have received education from college (undergraduate), 30 (29.4%) have gone to secondary school (up to standard XII), 25 (24.5%) have gone to primary school (up to standard V), and 14 (13.7%) have never enrolled in formal education (illiterate).

**Table 6:** Educational status of poisoning patients

Educational status	No. of patients
Graduate	31 (30.4%)
Secondary	30 (29.4%)
Primary	25 (24.5%)
Illiterate	14 (13.7%)
Postgraduate	2 (1.9%)
Total	102

Table 7 illustrates the causes of poisoning in our study population. The most common cause of poisoning was a non-impulsive suicidal intake of poison (n = 37, 36.4%), followed by impulsive suicidal intake (n = 29, 28.4%), and psychiatric illness leading to poisoning (n = 26, 25.5%) in the patients. The commonest factor in non-impulsive suicidal tendencies was stress, which was acute, episodic, and chronic. Out of all types of stress, the most common were those related to family (n = 14, 37.8%), finance (n = 11, 29.8%), relationships (n = 4, 10.8%), and office work (n = 3, 8.1%). The suicidal tendency in psychiatric illness was seen more in depression (n = 18, 69.3%), bipolar disorder (n = 6, 23.1%), and hallucinations (n = 2, 7.7%). 

**Table 7:** Cause of poisoning

S.N	Cause of poisoning (%)			No. of patients (n=102)
1	Impulsive suicidal (28.4%)			29
2	Non-impulsive suicidal (36.4%)	Stress-related (acute/episodic /chronic)	Family-related	14
			Financial related	11
			Relationship related	04
			Office work-related	03
			Unemployment related	03
			Education related	02
3	Accidental (8.8%)			09
4	Homicidal (0.9%)			01
5	Psychiatric illness (25.5%)	Depression		18
		Bipolar disorder		06
		Hallucination		02
Total				102

Most of the patients were suicidal (n = 92, 90.2%). Accidental poisoning was observed in nine (8.8%) individuals and homicidal in one (0.98%), and the difference is statistically significant (p<0.05). In most individuals, the place of poisoning was home (n = 93, 91.2%), where it was consumed with suicidal intent or by accidental exposure. The route of poisoning for nearly all individuals was oral, except in one case, which was by the injectable route. In our study, the timing of poisoning in the majority of patients was between 06:00 pm to 12:00 am (47%, n = 48) and 12:00 pm to 06:00 pm (31.4%, n = 32).

**Figure 1:**
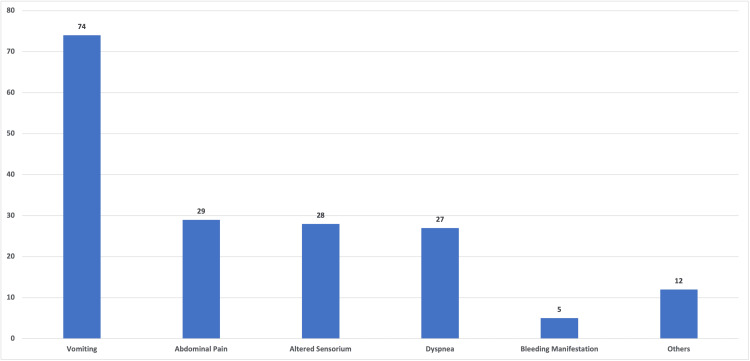
Frequency of the presenting complaints among the study population The X-axis represents the common symptoms, and the Y-axis represents the frequency of particular symptoms (number).

As shown in Figure 1, vomiting was the most common clinical manifestation, which was present in 74 (72.5%) of the individuals, followed by abdominal pain in 29 (28.4%), altered sensorium in 28 (27.45%), and difficulty breathing in 27 (26.4%) of the patients. Figure 1 represents the frequency of presenting complaints among the study population. The bleeding manifestation was noted in five (4.9%) of the individuals. In our study, 42 patients (41.2%) were initially admitted to other hospitals, 39 patients (38.2%) underwent gastric lavage, and 14 patients (13.7%) received an antidote. In our emergency department, 61 patients (59.8%) received gastric lavage, and 37 patients (36.3%) received an antidote.

Tables 8 and 9 describe the agents of poisoning, and the most common agent of poisoning was pesticide ingestion, accounting for 45 (44%) of the total cases. Organophosphorus contributed to the maximum number of poisoning patients (n = 31, 30.4% of the total cases of poisoning, and 68.8% of all pesticide poisoning cases). Prescribed drugs were the second-largest group (n = 19, 18.6%). Other common poisoning agents were rodenticides (n = 12, 11.7%), corrosives (n = 8, 7.8%), and aluminum phosphide (n = 3, 2.9%), and the difference in the distribution is statistically significant (p<0.001).

**Table 8:** Types of poisoning

Agents of poisoning	No. of patients (n=102)	P value
Pesticide	45 (44%)	<0.001
Prescribed drugs	19 (18.6%)	
Rodenticides	12 (11.7%)	
Corrosives	8 (7.8%)	
Aluminium phosphide	3 (2.9%)	
Others	15 (14.7%)	
Total	102	

**Table 9:** Types of various pesticide poisoning

S.N	Pesticide	No. of patients
1	Organophosphates	31 (68.8%)
2	Pyrethroids	4 (8.8%)
3	Paraquat	4 (8.8%)
4	Others	6 (13.3%)
	Total no of pesticide poisoning patients	45 (100%)

The complications and outcomes of organophosphorus poisoning are shown in Tables 10 and 11. Around 22 (70.9%) of the organophosphorus cases survived without any residual illness, whereas eight (25.8%) of them died during treatment and one (3.22%) patient did not consent to treatment (LAMA), and the difference in the distribution is statistically significant (p<0.001).

**Table 10:** The outcome of organophosphorus poisoning P value calculated through Fisher's exact test. LAMA: left against medical advice

Outcome (n=31)		P value
Survived	22	<0.001
Death	8	
LAMA	1	

**Table 11:** Complications in patients with organophosphorus poisoning

Complications	No. of patients (n=31)
Intermediate syndrome	5 (16.1%)
Hospital-acquired pneumonia	4 (12.9%)
Respiratory failure	8 (25.8%)

A total of 12 patients presented with a history of rodenticide ingestion. Around seven out of 12 patients (58.3%) developed hepatic complications. Of the patients, nine improved without any residual illness, while three patients (25%) died during treatment.

A total of eight patients presented with a history of corrosive poisoning. Although all patients survived, one patient required jejunostomy due to severe esophagitis and gastritis, while others did not have any residual illness.

Three patients presented with aluminum phosphide ingestion. One patient developed cardiogenic shock, and another developed dysrhythmia (ventricular tachycardia) and acute kidney injury (AKI). One patient survived, one patient died due to dysrhythmia, and one patient left against medical advice.

One patient presented with carbamate poisoning and died within one day of admission. Four patients presented with paraquat poisoning. All four patients had shortness of breath at the time of presentation. All patients had hepatic complications, and three of these four patients developed acute kidney injury (AKI). Three of the four patients (75%) died due to acute respiratory distress syndrome (ARDS), while one patient did not consent to treatment or LAMA.

Four patients presented with pyrethroid poisoning. All of them survived and were discharged without any residual illnesses. Other pesticides used included imidacloprid, clothianidin, and potash ingestion. Two patients had a history of unknown insecticide poisoning, of which one developed methemoglobinemia and died after five days of hospitalization.

Table 12 describes the prescribed medications for poisoning by overdose. Prescribed drugs were found to be the second most common method of poisoning (n = 19, 18.6%). Among these, benzodiazepines were the most commonly used agents (n = 6, 5.88% of all cases). The commonest presentation was an altered sensorium. During hospitalization, one patient developed respiratory depression and required mechanical ventilation. Among other drug groups (antipyretics and antiepileptics), four patients had a drug-induced liver injury (DILI), and two patients had prolonged prothrombin time. All the patients recovered eventually. 

**Table 12:** Agents used in poisoning with drugs

Drugs	Frequency
Benzodiazepine	6
Anti-psychotics	3
Antiepileptics	2
Antihypertensives	2
Antipyretics	3
Metformin	1
Amitriptyline	1
Deriphylline	1
Total	19

Out of all the poisoning cases, 71 (69.6%) recovered, 23 (22.54%) died, and eight of them were discharged with LAMA (7.84%), and the difference in the distribution is statistically significant (p<0.05). The mean duration of hospital stays for the patients was 6.4 days. Patients with paraquat poisoning were noted to have had an average hospital admission of 11.5 days.

Table 13 shows the poisoning of the study population with miscellaneous agents. Three alcohol poisoning cases were referred from other hospitals in view of ARDS, and all died with an average hospital stay of 3.6 days. Other natural flowers and fruits also contributed to this group with no mortality. Three unknown substances were leading to hospital admission, and two out of three patients succumbed to this unknown poisoning.

**Table 13:** Poisoning with miscellaneous agents

Type of poisoning	No. of patients	Complication	Average hospital stay (days)	Outcome
Alcohol	3	Seizure in one patient, hypoglycaemia in one patient, aspiration pneumonia in one patient	3.6	All three patients died
Yellow oleander	1	-	4	Survived
Calotropis	1	-	1	Survived
Datura	1	-	7	Survived
Metanil yellow	1	Haemolytic anaemia	7	Survived
Naphthalene ball	1	-	2	Survived
Turpentine yellow	1	-	5	Survived
Dettol	1	-	6	Survived
Unknown	3	-	2.3	One patient survived, and two patients died

Table 14 demonstrates the logistic regression analysis using the Wald test for the outcomes. None of the factors showed statistically significant results for the outcome of the patients. Notably, the odds ratios for male gender (0.368; 95% confidence interval (CI): 0.124, 1.089), upper-lower socioeconomic status (0.848; 95% confidence interval (CI): 0.225, 3.196), and pre-hospital treatment (0.705; 95% confidence interval (CI): 0.277, 1.794) demonstrated a trend towards increased chances of survival. 

**Table 14:** Logistic regression analysis of poisoned patients for outcome Logistical regression analysis using the Wald test.

Predictor	B	SE	Wald	Df	Sig.	Exp (B)	95% CI for Exp (B)
Age	-0.028	0.016	3.075	1	0.080	0.972	0.942, 1.003
Sex	-1.001	0.554	3.259	1	0.071	0.368	0.124, 1.089
Socioeconomic status	-0.165	0.677	0.059	1	0.808	0.848	0.225, 3.196
Pre-hospital treatment	-0.350	0.477	0.539	1	0.463	0.705	0.277, 1.794
Type of poison	0.288	1.333	0.047	1	0.829	1.333	0.098, 18.192

## Discussion

As per the systematic review and meta-analyses of all observational studies published in the English language from January 2010 to May 2020, pesticide poisoning is the most common type of poisoning, with an overall prevalence of 63% (two in every three cases of poisoning) and a prevalence of around 65% in the adult population and 22% in children in India [8].

Most of the cases were young adults in the age group 18 to 27 (50.98%), followed by the 30 to 39 age group. The incidence in other studies like S.K. Das et al. [14], Bhoopendra Singh et al. [15], and Mukul Joshi et al. [16] was similar, where most cases were from the age group 20 to 29. A higher incidence was observed in males compared to females (Table 15).

**Table 15:** Comparison of the most common age groups of patients with other studies

Studies	Sample size (n)	Most common age group (years)	Sex ratio (M/F)
Present study	102	18-27 (50.98%)	1.6
Das et al. 2005 [14]	306	20-29 (40 %)	1.14
Singh et al. 2006 [15]	325	20-29 (36 %)	2.3
Joshi et al. 2015 [16]	120	20-29 (56%)	1.1

The high incidence at this age is obvious because this age group is exposed to several determining factors of life in terms of studies, career, marriage, and other life settlements, which may lead to emotional and mental stress that causes the individual to seek methods of suicide.

According to a retrospective analysis, poisoning due to household products, according to phone calls received at the National Pesticide Information Centre (NPIC) at AIIMS, New Delhi, found that the commonest route of pesticide exposure was oral (95.6%). Household pesticides were commonly implicated (43.7%), followed by household cleaners (21.8%), thermometer mercury (5.2%), naphthalene balls (5%), antiseptics (3%), kerosene (2%), and paint thinner (2%). Miscellaneous products comprising camphor, silica gel, hair dye, nail polish remover, cosmetics, and adhesives were also involved in poisoning (17.1%) [17]. Findings from our study were similar to those elicited by Das et al. and Kiran et al., with pesticides, drugs, and rat-killer poison being the most commonly used in decreasing order.

A review of the literature demonstrated that the present study findings (Table 16) are consistent with those of Joshi et al., Singh et al., and Dash et al. [8,9,10]. Variations that arise among these studies must be carefully interpreted since they depend on the differences in occupation, levels of stress, economic backgrounds of the communities studied, the dose of poison consumed, the availability of medical facilities, and latency in care-seeking.

**Table 16:** Comparison of the nature of poisoning with other studies *Dash et al. (2005) [14] have not mentioned the nature of poisoning in their study population.

Nature of poisoning	Singh et al. 2006 [15] (n=325)	Joshi et al. 2015 [16] (n=120)	Present study (n=102)
Suicidal	224 (69%)	96 (80%)	92 (90.2%)
Accidental	91 (28%)	19 (15.8%)	09 (8.82%)
Homicidal	10 (3%)	05 (4.2%)	01 (0.98%)

Among patients with poisoning, the majority belonged to an urban area (n = 67, 65.7%), which contrasted with the findings by Joshi et al., where the majority of the cases (72%) were from rural areas and 28% of cases were from urban areas. The increased prevalence in the urban population may be due to increasing interpersonal conflicts, poor social support, and more financial distress, which were exacerbated in the background of the COVID-19 pandemic.

The small sample size of the study is the main limitation of the study. The recall method and greater reliability in history for the type of poisoning can also be considered another setback for the study. The toxin levels were not measured in all the patients, which might have been more objective. Bearing in mind that the pandemic added to both distress, financial losses, and footfall in the emergency department, this could have been attributed to the smaller sample size.

Through this study, we gained insight into the current burden of the problem that will further aid decision-making regarding the necessary corrective measures that are acutely needed in the community that the tertiary care centers serve. This will aid thereafter in curating the toxicology registry. The dynamic changes in the poisoning profile of the community with time require periodic evaluation of the registry thereafter, aiding early identification of trends among vulnerable populations. Focusing on training rural healthcare doctors at frequent intervals and involving toxicologists at the district level can help in the management of patients. Pushing for organic fertilizers and pesticides and the involvement of NGOs in banning highly toxic substances might help decrease the incidence of pesticide poisoning and fatalities.

## Conclusions

Poisoning is a commonly encountered emergency, often secondary to deliberate self-harm through the ingestion of toxic substances, although accidental encounters are not uncommon. The most common poisoning agents were pesticides, followed by drugs, rodenticide poison, corrosive agents, and aluminum phosphide. A sizable mortality rate was noted with organophosphate poisoning, in particular. Maintaining a toxicology registry is pertinent to the early identification of rising trends in particular communities or groups, especially in areas with clusters of vulnerable populations. The registry should be evaluated frequently to look for dynamic changes in the community for better management of patients. The government, NGOs, and institutions should come forward to improve mental health, use safe pesticides, decrease over-the-counter medications, and hire toxicologists at the district level.
